# Mediating effect of gastrointestinal symptoms on dietary behavior and quality of life in Chinese adults with chronic gastritis—a cross-sectional study

**DOI:** 10.3389/fmed.2023.1178897

**Published:** 2023-08-03

**Authors:** Litong Zhao, Feng Zhang, Dan Kuang, Dan Li, Jiai Yan, Ju Yang, Qinyue Wang, Yingyu Wang, Jing Sun, Yiran Liu, Ping Liu, Yanping Xia, Hong Cao

**Affiliations:** ^1^Wuxi School of Medicine, Jiangnan University, Wuxi, China; ^2^Nutritional Department, Affiliated Hospital of Jiangnan University, Wuxi, China; ^3^Clinical Assessment Center of Functional Food, Affiliated Hospital of Jiangnan University, Wuxi, China; ^4^Department of Endocrinology, Affiliated Hospital of Jiangnan University, Wuxi, China; ^5^Wuxi Center for Disease Control and Prevention, The Affiliated Wuxi Center for Disease Control and Prevention of Nanjing Medical University, Wuxi, China

**Keywords:** chronic gastritis, quality of life, dietary behavior, symptom, mediating effect

## Abstract

**Background:**

Chronic gastritis is accompanied by varying degrees of gastrointestinal symptoms, which affect people’s quality of life. The association between dietary behaviors and gastrointestinal symptoms of patients with chronic gastritis has been proved recently. However, no studies have been conducted to investigate the relationship between dietary behaviors, gastrointestinal symptoms, and quality of life.

**Methods:**

A cross-sectional survey of 176 patients diagnosed with chronic gastritis aged 18 to 65 years, comprising their information on demographic characteristics, dietary behaviors, gastrointestinal symptoms, and quality of life, was collected. A descriptive analysis and a correlation matrix were used to illuminate the characteristics of the subjects and bivariate correlation, respectively. The mediation model was analyzed using the PROCESS macros for SPSS.

**Results:**

Demographic characteristics were found to influence the symptoms, dietary behaviors, and quality of life of chronic gastritis patients; in particular, students categorized by occupation had higher levels of gastrointestinal symptoms and lower levels of quality of life and dietary behavior. The study variables were all pound related. We found that gastrointestinal symptoms played a partial mediating role between dietary behavior and both the physical components summary and mental components summary, and the ratios of mediating effects to the total effect on the physical components summary and mental components summary were 23.5% and 21.5%, respectively.

**Conclusion:**

Our survey discovered that dietary behavior, gastrointestinal symptoms, and quality of life were all pairwise related. The effect of dietary behavior on quality of life was partially mediated by gastrointestinal symptoms. These results may provide a novel perspective for medical staff in improving the quality of life of patients with chronic gastritis.

## 1. Introduction

Chronic gastritis (CG) is one of the most common life-long, serious, and insidious illnesses experienced by human beings. One may estimate that more than half of the world population have this disease to some degree and extent, indicating that many hundreds of millions of people worldwide may have chronic gastritis in one form or another (1). In China, chronic gastritis is the most prevalent digestive disorders (2); according to endoscopic diagnosis, the prevalence of CG was close to 90% (3). The clinical manifestations of CG are similar to the spectrum of digestive symptoms (4). Any chronic gastrointestinal conditions have been associated with lower quality of life (QOL) (5), including CG (6, 7). Prior studies (6, 8) have shown that demographic factors or gastrointestinal (GI) symptoms frequently influence the QOL of CG patients.

Clinically, changes in dietary habits and lifestyle adjustments are part of the treatment of CG. According to the 2022 China consensus on chronic gastritis (2), individual dietary and lifestyle changes are reasonable in terms of preventing and treating gastritis. When patients believe their symptoms to be related to food, they change their diet by avoiding the foods that they perceive as symptomatic (9). A recent study (10) found that different dietary behaviors or preferences were linked to different symptoms, proving the link between diet and GI symptoms in CG patients.

However, few studies have discussed whether dietary behavior affects the QOL of CG patients or whether symptoms play a role in the influence of dietary behavior on the QOL of CG patients. This study aims to explore the influencing factors of QOL in Chinese adult patients with CG. We speculate that in addition to demographic factors, QOL is also affected by GI symptoms and dietary behaviors, that is, higher GI symptoms and worse dietary behaviors are associated with lower QOL. In addition, we will also explore whether dietary behavior may affect quality of life by causing GI symptoms.

## 2. Materials and methods

### 2.1. Participants and recruitment

A cross-sectional design was conducted in this study. A convenient sample of 176 patients with CG was included. Participants were recruited from the gastroenterology department and gastroenteroscopy center of a level III hospital in Wuxi, China. Participants were chosen based on the following criteria: (a) aged 18–65; (b) having the endoscope examination and reaching the pathological diagnostic criteria of non-atrophic gastritis; (c) all consecutive patients with gastrointestinal symptoms for a week or longer; (d) volunteering to participate in the questionnaire survey. Exclusion criteria included: (a) subjects who reported having other digestive system diseases such as peptic ulcer, pancreatitis, hepatitis, and cirrhosis; (b) having serious heart, brain, liver, kidney, or hematopoietic system dysfunction or other serious diseases; (c) having a severe mental illness (such as schizophrenia, depression, and anxiety); (d) food or drug allergies and pregnancy or breastfeeding; (e) taking a medication that had caused side effects of gastrointestinal symptoms for the past 3 months.

The sample size was 5–10 times the number of variables using the Kendall sample size calculation method. This study included 30 survey variables, multiplied by 5, and 20% of the incorrect questionnaires were taken into account. The total number of cases in the sample was 180.

### 2.2. Ethics and consent

The study was approved by the medical ethics committee of the Affiliated Hospital of Jiangnan University (LS2021012) and was performed in accordance with the principles of the Helsinki Declaration of 1964. Moreover, it was registered at the Chinese Clinical Trials Registry (http://www.chictr.org.cn/index.aspx Identifier: ChiCTR2200062781). All patients signed an informed consent before completing the measures.

### 2.3. Data collection

The demographic characteristics included sex: male or female; age: 18–24 years, 25–34 years, 35–44 years, 45–54 years, 55–64 years; Education: primary (≤9 years), secondary (9–12 years), some college (13–17 years), college graduate or higher (≥18 years); body mass index (BMI): BMI was defined as the body weight (in kilograms) divided by the square of the body height (in meters); occupation: student, staff, retired, others; chronic diseases history: gastric chronic diseases, others, none; smoking and drinking history: yes (more than one cigarette per day for more than a year or/and drinking more than 30 g a day for more than 6 months) or no.

### 2.4. Dietary behavior

A survey of dietary behaviors was conducted using the dietary behaviors scale (10, 11). The scale comprises 8 items: eating slowly, nutrition combination, regular mealtime, eating breakfast, overeating, spicy food, raw or cold food, and strong tea/coffee. The items were assessed using a five-point Likert-type scale (1 = never, 5 = always), with inverted ratings for “overeating,” “spicy and stimulating food,” “too cold or leftover food,” and “strong coffee and tea.” The higher the scores, the better the dietary behavior of the participants.

### 2.5. GI symptoms

With the clinical experience of clinical digestive experts, we evaluated five common symptoms: stomachache, belching, acid reflux, gastric distention, and early satiety. Each symptom was assessed on a four-point Likert scale, ranging from no discomfort to very severe discomfort. The higher the scores, the more severe the symptoms.

### 2.6. The simplified Chinese (mandarin) version of the SF-36 (CSF-36)

The CSF-36 showed good validity and reliability in the general Chinese population (12). Physical functioning (PF), role-physical (RP), bodily pain (BP), general health (GH), vitality (VT), social functioning (SF), role-emotional (RE), and mental health are among the eight domains represented by the CSF-36’s 36 items (MH). A physical components summary (PCS) and a mental components summary (MCS) can be created from these domains (6, 13). The score conversion formula is as follows: conversion score = (actual score—the lowest possible score in this aspect)/(the difference between the highest possible score and the lowest possible score in this aspect) × 100. The higher the score, the better the quality of life.

### 2.7. Quality control

All interviewees were medical professionals undergoing unified and standardized training to lower measurement variance. The participants were instructed to complete the questionnaire after giving their permission. The medical staff conducted the survey by interview, read the questions, and filled out the questionnaire under the responses. The participants also filled out the questionnaire on their own initiative or at their request.

### 2.8. Statistical methods

SPSS 25.0 was used to analyze the data, and a two-sided value of *p* <0.05 was considered to be statistically significant. Categorical variables (demographic characteristics) were presented using frequency and percentage. Continuous variables (dietary behavior, GI symptoms, SF-36) were presented using means, standard deviation, and range. Distributed differences of categorical variables were assessed using an independent sample *t*-test (sex, smoking, and drinking history) or one-way ANOVA (age, education, BMI, occupation, chronic diseases history). The association between dietary behaviors, GI symptoms, and QOL was examined using Pearson correlation. Hierarchical regression was used to examine the association between dietary behaviors, symptoms, and quality of life. After adjusting for demographic factors, the hierarchical regression analysis was completed using Hayes’ test steps, and the mediation effect test was run using the bootstrap technique 5,000 times (14).

## 3. Results

### 3.1. Participant characteristics

A total of 180 questionnaires were distributed and recovered, and 176 questionnaires met the criteria. Table 1 presents the participants’ demographic and clinical characteristics. Most participants were female (51.7%). The mean age was 39 (39.1 ± 13.2), and 23.3% of them ranged from 18 to 25 years old. In all, 50% of the participants had attained a college education, and 22.2% had been college graduates or attained a higher education. The participants included 91 (51.7%) employees, 46 (26.1%) students, 17 (9.7%) retirees, and some independent contractors and jobless individuals. The majority of the participants (63.1%) had a normal BMI, but 31.8% were overweight; 22.2% were students, and 16.5% were medical workers; 26% had a history of stomach problems, such as gastritis, and 11% had a chronic disease, such as hypertension or diabetes. Moreover, 22.2% of the respondents had a history of smoking or drinking.

**Table 1:** Demographic characteristics of the sample (*N* = 176).

Characteristics	Participants [*N* (%)]
*Sex*	
Male	85 (48.3%)
Female	91 (51.7%)
*Education*	
Primary (≤9y)	16 (9.1%)
Secondary (9–12y)	33 (18.8%)
Some college (13–17y)	88 (50%)
College graduate or higher (≥18y)	39 (22.2%)
*Age, y*	
18–25	41 (23.3%)
25–34	27 (15.3%)
35–44	38 (21.6%)
45–54	43 (24.4%)
55–64	27 (15.3%)
*BMI, kg/m^2^*	
Underweight (<18.5)	9 (5.1%)
Normal weight (18.5–23.9)	111 (63.1%)
Overweight (>24)	56 (31.8%)
*Occupation*	
Student	46 (26.1%)
Staff	91 (51.7%)
Retired	17 (9.7%)
Others	22 (12.5%)
*Chronic diseases history*	
None	111 (63%)
Gastric diseases	46 (26%)
Others	19 (11%)
*Smoking and drinking history*	
Yes	39 (22.2%)
None	137 (77.8%)

### 3.2. Symptoms, dietary behaviors, PCS, and MCS scores

The following data analysis results show the status of GI symptoms, dietary behaviors, and QOL scores of the CG patients (Table 2): the mean symptom score was 4.2, indicating that everyone had at least two symptoms on average. The mean dietary behaviors score was 28.9, which was greater than 20 (total score/2), indicating that the dietary behaviors were relatively good. Furthermore, the PCS score was 310.7, which was higher than the MCS score of 286.3.

**Table 2:** Scores of symptoms, signs, dietary behaviors, and CSF-36 of the participants.

Symptoms and signs	M ± S (range)	Dietary behaviors	M ± S (range)	CSF-36	M ± S (range)
Stomachache	0.9 ± 0.6 (0–3)	Eating slowly	3.3 ± 1.0 (1–5)	PF	91.8 ± 14.3 (0–100)
Hiccup/belching	1.0 ± 0.7 (0–3)	Nutrition combination	3.5 ± 1.0 (1–5)	RP	84.8 ± 29.9 (0–100)
Acid reflux	1.0 ± 0.7 (0–3)	Regular mealtime	3.9 ± 0.9 (1–5)	BP	72.0 ± 16.0 (12–90)
Gastric distention	0.9 ± 0.6 (0–3)	Eating breakfast	4.2 ± 1.0 (1–5)	GH	62.1 ± 18.0 (20–100)
Early satiety	0.5 ± 0.6 (0–2)	Overeating	3.6 ± 0.8 (1–5)	VT	57.3 ± 11.4 (25–75)
Symptom score	4.2 ± 2.1 (1–11)	Spicy food	3.2 ± 0.9 (1–5)	SF	82.1 ± 16.6 (33.3–100)
—	—	Raw or cold food	3.6 ± 0.9 (1–5)	RE	76.6 ± 35.9 (0–100)
—	—	Strong tea/coffee	3.6 ± 1.0 (1–5)	MH	70.2 ± 14.4 (32–100)
—	—	Dietary behaviors	28.9 ± 4.2 (17–40)	PCS	310.7 ± 54.4 (137–387)
—	—	—	—	MCS	286.3 ± 58.6 (113.4–367)

Values are expressed as means (M) ± standard deviation (S) and range. PF, physical functioning; RP, role-physical; BP, bodily pain; GH, general health; VT, vitality; SF, social functioning; RE, role-emotional; MH, mental health; PCS, physical components summary (weighted sum of PF, RP, BP, and GH); MCS, mental components summary (weighted sum of VT, SF, RE, and MH).

### 3.3. Factors with impacts on dietary behaviors, GI symptoms, PCS, and MCS

The influencing factors on the research variables were analyzed based on demographic characteristics (Table 3), and the results are as follows: (a) in terms of the GI symptom score, chronic diseases and occupation had a significant effect (*p* < 0.05); that is, a history of stomach problems, and the student group had a higher score, with the symptoms being relatively more severe. (b) Dietary behaviors: educational level, age, and occupation had a significant impact on dietary behavior scores (*p* < 0.05); the higher the education level and the younger the participant, the worse the dietary behavior. (c) PCS: PCS was affected by different age groups and occupations (*p* < 0.05). People between the ages of 25 and 54–65 had a lower PCS score, corresponding to students and retirees in occupations. (d) MCS: Age, education level, occupation, and smoking and drinking history all had a significant impact on MCS (*p* < 0.05), with those under 25 years old, with a master’s degree or higher, students, and those who do not smoke or drink scoring lower. (e) Sex and BMI had no significant effect on dietary behavior, symptoms, PCS, and MCS in our study.

**Table 3:** Factors with impact on symptoms, signs, dietary behaviors, PCS, and MCS.

Characteristics		Symptoms (M ± S)	PCS (M ± S)	MCS (M ± S)	Dietary behaviors (M ± S)
Sex	Female	4.3 ± 2.2	309.6 ± 57.9	279.1 ± 61.6	28.9 ± 4.8
	Male	4.1 ± 2.0	312.0 ± 50.7	294.0 ± 54.5	28.9 ± 3.4
*t*/*p*		0.7/0.482	−0.3/0.762	−1.694/0.092	0.1/0.934
Education	Primary	4.1 ± 2.2	296.9 ± 59.0	291.4 ± 44.4^ab^	31.5 ± 4.2^ab^
	Secondary	3.7 ± 1.8	318.4 ± 37.5	316.1 ± 37.6^a^	30.2 ± 3.5^ab^
	Some college	4.2 ± 2.2	312.8 ± 55.93	282.3 ± 64.1^b^	28.8 ± 4.2^b^
	College graduate or higher	4.6 ± 2.2	305.2 ± 61.2	267.7 ± 57.2^b^	27.1 ± 4.0^c^
*F*/*p*		1.0/0.393	0.7/0.528	4.6/0.004	6.1/0.001
Age	<25	4.9 ± 2.3	298.4 ± 64.4^a^	250.6 ± 66.9^a^	26.7 ± 3.7^b^
	25–34	4.5 ± 2.2	299.4 ± 65.2^ab^	269.0 ± 70.7^ab^	26.7 ± 4.0^b^
	35–44	3.9 ± 1.9	329.7 ± 40.6^b^	312.3 ± 44.6^b^	29.2 ± 3.2^b^
	45–54	3.7 ± 1.9	322.5 ± 43.2^b^	302.1 ± 42.0^b^	30.1 ± 4.2^b^
	55–65	4.0 ± 2.3	295.3 ± 50.2	295.7 ± 41.6^b^	32.0 ± 3.6^a^
*F*/*p*		2.287/0.062	3.178/0.015	8.441/0	11.440/0
Occupation	Student	5.0 ± 2.3^a^	291.5 ± 67.6^a^	245.36 ± 64.8^a^	26.4 ± 3.9^a^
	Staff	3.9 ± 2.0^b^	322.7 ± 46.7^b^	302.0 ± 50.6^b^	29.1 ± 3.9^b^
	Retired	3.7 ± 2.3^b^	292.8 ± 48.4^b^	295.2 ± 43.2^b^	33.1 ± 3.9^b^
	Others	4.0 ± 2.0^ab^	315.3 ± 44.7^b^	299.8 ± 47.1^b^	30.0 ± 3.2^b^
*F*/*p*		2.9/0.038	4.3/0.006	12.1/0	14.2/0
BMI	Lean	3.9 ± 2.3	318.3 ± 45.1	277.2 ± 72.7	29.1 ± 4.5
	Normal	4.4 ± 2.1	307.2 ± 59.5	279.4 ± 64.0	28.7 ± 4.1
	Overweight	3.9 ± 2.1	316.6 ± 44.3	301.3 ± 40.4	29.3 ± 4.3
*F*/*p*		1.3/0.266	0.6/0.526	2.8/0.065	0.5/0.624
chronic diseases history	Gastric chronic diseases	4.8 ± 2.3^a^	308.5 ± 59.8	278.4 ± 64.6	29.1 ± 4.4^ab^
	None	4.1 ± 2.1^ab^	312.2 ± 52.4	288.3 ± 58.9	28.5 ± 4.1^a^
	Others	3.3 ± 1.7^b^	307.8 ± 54.6	293.5 ± 38.6	30.8 ± 3.7^b^
*F*/*p*		3.6/0.029	0.104/0.901	0.6/0.541	2.6/0.076
Smoking and drinking	Yes	4.5 ± 1.6	315.4 ± 49.7	312.7 ± 37.8^a^	28.7 ± 3.6
	None	4.1 ± 2.2	309.5 ± 55.7	279.2 ± 61.2^b^	29.0 ± 4.3
*t*/*p*		−1.3/0.212	−0.6/0.557	−4.1/0	0.4/0.705

M, mean; S, standard deviation. The same letter of superscript in the data of the same categorical variable means that the difference is not significant (*p* > 0.05), and different letters mean that the difference is significant (*p* < 0.05). PCS, physical components summary (weighted sum of PF, RP, BP, and GH); MCS, mental components summary (weighted sum of VT, SF, RE, and MH).

### 3.4. Bivariate correlations among dietary behaviors, GI symptoms, PCS, and MCS

The bivariate correlation analysis (Table 4) revealed a pair-to-pair correlation between GI symptoms, dietary behavior, and PCS (MCS). Dietary behavior was negatively correlated to symptoms (*r* = −0.3, *p* < 0.01) while positively related to PCS (*r* = 0.3, *p* < 0.01) and MCS (*r* = 0.3, *p* < 0.01). Symptoms were negatively related to PCS (*r* = −0.4, *p* < 0.01) and MCS (*r* = −0.4, *p* < 0.01). Furthermore, PCS was moderately related to MCS (*r* = 0.7, *p* < 0.01).

**Table 4:** Correlation analysis of symptoms, dietary behaviors, PCS, and MCS in patients with chronic gastritis.

	1	2	3	4
1. Symptoms	1			
2. Dietary behaviors	−0.3^**^	1		
3. PCS	−0.4^**^	0.3^**^	1	
4. MCS	−0.4^**^	0.3^**^	0.7^**^	1

^**^p < 0.01. PCS, physical components summary (weighted sum of PF, RP, BP, and GH); MCS, mental components summary (weighted sum of VT, SF, RE, and MH).

### 3.5. Analysis of the mediating effects of gastrointestinal symptoms

The PROCESS Model 4 developed by Hayes (14) was used for regression analysis (Table 5). After controlling for covariables (age and occupation), equation 1 was established with PCS as the dependent variable and dietary behavior as the independent variable. The dependent variable in equation 2 was symptoms, and the independent variable was dietary behaviors. Equation 3 was established with PCS as the dependent variable and dietary behavior and symptoms as the independent variable. The findings revealed that dietary behavior could positively predict PCS (*β* = 0.3, *p* < 0.05) and negatively predict symptoms (*β* = −0.2, *p* < 0.05). PCS was significantly predicted by dietary behaviors (*β* = 0.2, *p* < 0.05) and symptoms (*β* = −0.3, *p* < 0.05). The addition of symptoms to equation 3 based on equation 1 improved the explanatory degree, implying that symptoms acted as a moderator in the influence of dietary behaviors on PCS. The effect of GI symptoms on dietary behaviors and MCS was investigated (Table 6). The bootstrap 95% CI of the direct and indirect effects of dietary behavior on PCS did not contain 0, indicating that symptoms played a partial mediating role between dietary behaviors and PCS, with a mediating effect value of 0.9, accounting for 23.5% of the total effect value (3.9). The same method was used to examine the mediating effect of symptoms on the influence of dietary behaviors on MCS, and the findings revealed that symptoms played a partial mediating role between dietary behaviors and MCS, with a mediating effect value of 0.8, accounting for 21.5% of the total effect value (3.6).

**Table 5:** Regression analysis on PCS (MCS).

	Equation 1		Equation 2		Equation 3		Equation 4		Equation 5		Equation 6	
	*β*	*t*	*β*	*t*	*β*	*t*	*β*	*t*	*β*	*t*	*β*	*t*
Dietary behaviors	0.3	3.7^***^	−0.2	−2.9^**^	0.2	2.9^**^	0.3	3.3^***^	−0.2	−2.5^*^	0.2	2.6^**^
GI symptoms					−0.3	−4.1^***^					−0.3	−3.7^***^
*R*^2^	0.2		0.1		0.2		0.3		0.1		0.3	
*R*^2^_adj_	0.1		0.1		0.2		0.2		0.1		0.3	
*F*	4.1^***^		2.5^*^		5.9^***^		5.0^***^		2.0^*^		6.0^***^	

Boot SE boot CI lower limit and boot CI upper limit refer to the standard error of the indirect effect estimated by the deviance-corrected percentile bootstrap method and the lower limit and upper limit of 95% CI, respectively. *p < 0.05, ^**^p < 0.01, ^***^p < 0.001.

**Table 6:** Mediating effects of symptoms on dietary behaviors and PCS (MCS).

	Path	Effect	Boot SE	Bootstrap 95% CI		Effect size/%
				BootLLCI	BootULCI	
Total effect 1	Dietary behaviors → PCS	3.9	1.0	1.9	6.0	
Direct effect 1	Dietary behaviors → PCS	3.0	1.0	1.0	5.0	76.5
Indirect effect 1	Dietary behaviors → symptoms → PCS	0.9	0.4	0.2	1.8	23.5
Total effect 2	Dietary behaviors → MCS	3.6	1.1	1.4	5.7	
Direct effect 2	Dietary behaviors → MCS	2.8	1.1	0.7	4.9	78.5
Indirect effect 2	Dietary behaviors → symptoms → MCS	0.8	0.4	0.1	1.6	21.5

Boot SE boot CI lower limit and boot CI upper limit refer to the standard error of the indirect effect estimated by the deviance-corrected percentile bootstrap method and the lower limit and upper limit of 95% CI, respectively. PCS, physical components summary (weighted sum of PF, RP, BP, and GH); MCS, mental components summary (weighted sum of VT, SF, RE, and MH).

## 4. Discussion

To assess the quality of life of patients with CG, more studies are currently being conducted that focus on the research of demographic characteristics and symptoms (6, 8). The effect of dietary behavior on QOL is rarely explored, although it constitutes a common measure in the prevention and treatment of CG (15). The link between dietary behavior and symptoms has been studied in recent years (10, 16, 17). We evaluated patients with symptomatic CG to better understand the potential roles of dietary behavior and symptoms in QOL.

The average symptom score was 4.2, indicating that participants with CG had at least two symptoms on average. The average age of participants was 39.1, which corresponded to the general trend of CG patients being younger (18). Additionally, due to the proximity of our hospital to the university, a relatively large number of undergraduate and postgraduate students (26.1%) participated in the survey. This group of students showed greater symptom scores and lower dietary behavior and quality of life scores. Excessive mental stress and poor dietary behavior are frequent contributing factors to CG (19). Anxiety (20) and dietary behaviors (16, 17) were often associated with GI symptoms, and positive psychological wellbeing and health behavior could moderate GI symptoms and result in a better QOL (21). Chinese undergraduate and postgraduate students have a heavier academic burden and poorer ability to regulate emotional and life stress than other groups, which may be the main reason for the lower QOL of these students. These results suggest that in future research, we should pay more attention to CG in student populations. Additionally, we found that patients with a history of digestive disorders had more severe symptoms. This may be because people with a lengthy history of stomach issues are more sensitive to symptoms. While body function decreases with age and gastrointestinal function in CG patients reduces physiological function and everyday activities, thus affecting their quality of life, retired and elderly individuals had lower PCS scores. This is because, at the same time that body function declines with age, both factors contribute to the body’s tendency to become less functional (22). Higher MCS scores have been found among individuals with a history of smoking and drinking, and we hypothesized that these behaviors would in some situations be beneficial for emotional release.

The bivariate correlation analysis revealed a pair-to-pair correlation between GI symptoms, dietary behavior, and PCS (MCS). In addition, the mediation model showed that GI symptoms played a partial mediating role between dietary behavior and both PCS and MCS, and the ratios of the mediating effects to the total effect were 23.5 and 21.5%. The findings suggested that better dietary behavior was associated with lighter GI symptoms, which, in turn, was associated with better QOL in both PCS and MCS. With fewer and milder GI symptoms, CG patients can endure less pain and have a more relaxed life. The mediating effects in our study accounted for 23.5% and 21.5% of the variance. It is possible that some variables we did not take into account occupied more weight, such as the covariates we accounted for, age and occupation, and the quantity of unhealthy diets that we did not consider in our study. This study can provide medical workers with a recommendation for patients with symptomatic CG; indeed, the evaluated and corrected dietary behavior of patients can reduce symptoms and improve their quality of life.

Our survey discovered that dietary behavior, GI symptoms, and QOL are all pairwise related. The effect of dietary behavior on QOL is partially mediated by GI symptoms. These results may provide a novel perspective for medical staff in improving the quality of life of patients with CG. There are some limitations in the study. First, the study was a cross-sectional survey, which means it can only offer causal hypotheses and cannot carry out extensive causal verification. Second, the results could not reflect the entire target population due to the small sample size, and age subgroups have not been examined. Third, incorporating food frequency and quantity into the reference range may enhance the influencing variables. In the future, we will conduct a multi-center study, adopt probability sampling to increase the sample size, and focus on the quality of life of patients with chronic gastritis of different ages to strengthen the research results.

## Data availability statement

The original contributions presented in the study are included in the article/supplementary material, further inquiries can be directed to the corresponding author.

## Ethics statement

Written informed consent was obtained from the individual(s) for the publication of any potentially identifiable images or data included in this article.

## Author contributions

FZ and DK: conception and design. LZ and FZ: analysis and interpretation of the data and drafting of the paper. LZ, DL, JiY, JuY, QW, YW, JS, YL, and YX: data collection and its critical revision for intellectual content. HC: supervision, project administration, and writing—reviewing and editing. All authors contributed to the article and approved the submitted version.

## Funding

We are grateful to the Natural Science Foundation of Jiangsu Province (BK20210060 and SBK2022023007); Scientific Research Project of Jiangsu Commission of Health (M2021055); the funding for Leading Talents and Advanced Talents in Medical and Health Profession in Wuxi Taihu Lake Talent Plan; Science and Technology Program Project of Jiangsu Market Supervision and Administration (KJ2022028); Jiangsu Scientific Research Project of Elderly Health (LK2021035); Jiangsu Scientific Research Project of Women’s and Children’s Health (F201741); Scientific Research Project of Wuxi Commission of Health (ZZ003 and Q201762); Wuxi Scientific and Technological Development Project (N20192024, N20191001, and Y20212001); and Translational Medicine Research Program of Wuxi Translational Medicine Center (2020ZHYB08).

## Conflict of interest

The authors declare that the research was conducted in the absence of any commercial or financial relationships that could be construed as a potential conflict of interest.

## Publisher’s note

All claims expressed in this article are solely those of the authors and do not necessarily represent those of their affiliated organizations, or those of the publisher, the editors and the reviewers. Any product that may be evaluated in this article, or claim that may be made by its manufacturer, is not guaranteed or endorsed by the publisher.
